# Nameability effects and short-term memory limitations on order perception and enumeration of brief sounds

**DOI:** 10.1371/journal.pone.0304913

**Published:** 2024-06-20

**Authors:** Anna-Maria Psarompa, Fotios Fotiadis, Argiro Vatakis

**Affiliations:** 1 Faculty of Medicine, University of Athens, Athens, Greece; 2 Department of Psychology, Multisensory and Temporal Processing Laboratory (MultiTimeLab), Panteion University of Social and Political Sciences, Athens, Greece; Bournemouth University, UNITED KINGDOM

## Abstract

Research has shown that perceiving the order of successive auditory stimuli could be affected by their nameability. The present research re-examined this hypothesis, using tasks requiring participants to report the order of successively presented (with no interstimulus gaps) environmental (i.e., easily named stimuli) and abstract (i.e., hard-to-name stimuli) sounds of short duration (i.e., 200 ms). Using the same sequences, we also examined the accuracy of the sounds perceived by administering enumeration tasks. Data analyses showed that accuracy in the ordering tasks was equally low for both environmental and abstract sounds, whereas accuracy in the enumeration tasks was higher for the former as compared to the latter sounds. Importantly, overall accuracy in the enumeration tasks did not reach ceiling levels, suggesting some limitations in the perception of successively presented stimuli. Overall, naming fluency seemed to affect sound enumeration, but no effects were obtained for order perception. Furthermore, an effect of each sound’s location in a sequence on ordering accuracy was noted. Our results question earlier notions suggesting that order perception is mediated by stimuli’s nameability and leave open the possibility that memory capacity limits may play a role.

## Introduction

Perceiving the temporal order of acoustic events is affected by stimulus parameters and presentation characteristics. Aaronson [1] suggested that the accuracy of recalling acoustic sequences is primarily affected by the length of the intervals separating the sounds, during which the stimuli are being recognized and processed. Subsequent studies showed that stimulus duration also affects temporal order perception of sound sequences (e.g., [2,3]). For example, Thomas et al. [2]. found that when it comes to vowels, the ability to perceive their presentation order severely decreases for durations shorter than 125 ms. Additionally, Warren et al. [3] showed that participants perceived the order of sequences of verbal auditory stimuli (i.e., words of 200 ms each with no interstimulus interval) with higher accuracy as compared to sequences of abstract (artificial) sounds (i.e., tones, hisses, and buzzes) of the same duration. Further comparison of the results from Warren et al. with those from Thomas et al. suggested that familiarity with the stimuli presented could also influence the perception of temporal order [2].

Apart from familiarity, the study of Warren et al. [3] also raised an issue regarding order processing of abstract auditory stimuli. That is, the increased difficulty in order perception of acoustically less complex stimuli (i.e., abstract sounds compared to verbal stimuli) contradicted the widespread belief that, after sensory registration, acoustic stimuli are decomposed to their components, and recognized based on the sequence of those components [4]. This finding led to a series of experiments, aiming to shed light on the factors influencing the perception of temporal order of abstract and verbal sounds. Firstly, Warren and Obusek [5] investigated whether response format could play a role in participants’ behavior by repeating Warren et al.’s experiment and manipulating response format (i.e., verbal response as compared to card sorting, where cards contained each stimulus’ “name”). Results showed that responding by physically sorting cards allowed participants to correctly report the sequence of stimuli lasting up to 300 ms, whereas for verbal reports the same limit was at 670 ms. The authors, thus, suggested that responding with cards allowed participants to consider the order of the stimuli without having to maintain the answer in working memory before articulating it (i.e., smaller working memory load). Secondly, Warren [6] investigated the least number of sounds that should be presented in a sequence to eliminate the possibility of response strategies (e.g., one could derive the order of sounds solely based on the ones presented at the beginning or end of a sequence). This led him to the conclusion that order perception should not be tested with sequences of less than four sounds given that participants recognized the first and last elements of a sequence more accurately compared to the sounds in the middle of the sequence. Thus, using less than four sounds would measure more the perception of onset and termination rather than perception of order, by allowing participants to infer the order of sounds by detecting the first and last elements of a two- or three-item sequence [7]. Furthermore, other researchers have also noted perception deficits for elements in the middle of a sequence [8–10], and have posited some kind of short-term memory limitation.

It was additionally argued that the perception of temporal order could be affected by melodicity. Specifically, Warren and Obusek [5] conducted an experiment with a condition where two sounds (i.e., a high and a low note) had frequencies that allowed for merging into a melodic conjunction. Results showed that when the two aforementioned sounds were presented in succession, participants exhibited higher accuracy in reporting the order of stimuli in a sequence compared to trials where the two sounds were not presented in succession. The authors posited a mechanism driven by evolution allowing participants to conceive two sounds as a pattern—similarly to speech sounds—, while at the same time “excluding” [5] irrelevant sounds. This would mean that the perception of characteristics that differentiate two sequences (e.g., pitch and quality) is a process distinct from the perception of the order of individual elements within a sequence.

This notion of distinct processes was further refined by Warren [11], who showed that participants could identify whether two abstract sound sequences were identical even when the individual sounds composing them were as short as 5 to 10 ms—while their order could not be perceived. This supported the hypothesis that “identification of patterns precedes identification of the component items and their order” [5, p. 90]. Thus, Warren and Ackroff [12] later posited two processes for order perception. The first, namely Holistic Pattern Recognition (hereafter HPR), concerned identification of patterns based on pitch and quality, and is recruited at durations of only a few milliseconds. The second, namely Direct Identification of Components and their Order (hereafter Direct ICO), regards identification of individual components and of their order upon presentation. It was hypothesized that Direct ICO can be recruited only if elements in a sequence last 200 ms or more, a time limit that purportedly resulted from the fact that to successfully perceive the order of components one would have to name each individual component—attach a verbal label to it [13]. This hypothesis corroborated previous results by Warren et al. [3], namely that perception of the order of verbal stimuli (i.e., words lasting 200 ms, thus recruiting the Direct ICO process) was more accurate and faster than non-verbal stimuli since the sound and its name coincided, something that was not true for abstract sounds [13].

Warren and Ackroff [12] suggested that the purpose of each of these mechanisms is not exclusive: both Direct ICO and HPR can be used to identify patterns of sounds as well as to perceive the order of components within them. The mechanism used is predisposed by two factors: 1) the coherence of stimuli within the sequence, where coherent sounds such as speech or music are most likely to be processed by HPR, while non–related sounds within a sequence tend to be processed by Direct ICO [13] and 2) stimulus duration, where the shorter the stimuli the higher the possibility of HPR processing, while longer stimuli are most probably processed by Direct ICO [12]. Importantly, the scope of each mechanism can be expanded by familiarity with the stimuli or training with a task. Particularly, Direct ICO allows for identification of order as well as for pattern recognition and can be used for incrementally shorter item durations as familiarity increases [12]. Also, HPR allows both for perception of order and patterns of coherent stimuli, but with training, this mechanism can also be recruited for sequences of unrelated sounds [13]. The aforementioned distinction was further enriched by suggesting that HPR is an earlier-stage mechanism that could be used by non-human animals [13], while Direct ICO was suggested to be an evolutionarily later process given that it depends on the use of verbal labels and allows for decomposition of longer unfamiliar stimuli via naming [13].

If Direct ICO is indeed a mechanism recruited with stimuli lasting 200 ms or more, then higher nameability for brief acoustic stimuli should facilitate the perception of their order, given that identification of components depends on the availability of stimuli names [13].Literature has also linked auditory temporal perception to language, through the study of relevant disorders such as aphasia (e.g.[14,15]) and developmental dysphasia [16]. Despite the existing evidence for a connection between language and time perception, no study has revisited the hypothesis posited by Warren et al. [3], that the ability in naming the presented auditory stimuli supports our ability in deciphering their order of appearance. Exploring the mechanisms of order perception and their purported interaction with language processes, might elucidate our understanding of the deficit underlying language disorders.

To test this hypothesis, we used a within-subjects design and compared participants’ accuracy in reporting the order of easy-to-name sound stimuli (environmental sounds; cf. verbal stimuli in [3]) with accuracy in reporting the order of abstract sounds (identical to those used in [3]). Unlike previous studies on the matter, we presented the sequences once and not in loops (cf. [3,5,11,12,17]; but see [3,5]), to control for participants’ exposure to the stimuli and given findings supporting that sequence repetition results in a difficulty to demarcate the boundaries of a sequence, making it harder to initiate encoding of order [9]. We also modified the response procedure with participants providing their response by successively clicking on the names of the stimuli (which they had previously selected) presented on the screen. We also investigated whether difficulty in order perception might also coincide with difficulty in the perception of the stimuli per se. To this end, prior to the ordering tasks, we administered enumeration tasks using the same sequences from the two sound categories (i.e., abstract and environmental), hence any difficulty in perceiving individual stimuli should be reflected in counting accuracy. We anticipated that if naming fluency affects order perception, order reporting accuracy should be higher in the environmental sound sequences compared to the abstract sound sequences. We also hypothesized that if difficulty in order reporting is mediated by difficulty in the perception of individual stimuli, participants should also exhibit lower counting accuracy in the abstract compared to the environmental sound sequences.

## Method

### Participants

An a priori power analysis was performed on G*Power software (version 3.1.9.7) to indicate the appropriate sample size for each task [18]. For that purpose, we utilized effect sizes of sound type from a pilot study we conducted and kept the highest recommended sample size. For the ordering tasks, effect size was *d* = 1.41, and for the enumeration tasks the effect size was *d* = 0.72. We set significance at α = 0.05 and power at 1-β = 0.95, and the recommended sample size was 28 participants. In total, 40 students were recruited before reaching the appropriate sample size. There was an a priori elimination criterion regarding performance in the ordering tasks: participants who did not select four different stimulus labels (but instead selected twice or more the same, one label) for five trials or more, were considered not to follow experimental instructions. Four participants exhibited this behavior and were, thus, removed from our sample. One of those four participants also had a psychiatric diagnosis, which was an additional exclusion criterion. We also discarded data from five participants given their extensive music experience (i.e., more than 10 years of musical training or systematic playing of musical instruments in the last five years), to ensure that enhanced acoustic order perception could not be attributed to musical expertise (see also Discussion section on this issue). We, finally, discarded data from one participant who exhibited zero accuracy in all the tasks, as well as participants with a history of neurological or psychiatric illness (i.e., two participants with a neurologic condition, as well as one with a psychiatric diagnosis who also did not meet the label-selection criterion and is mentioned above) to deter any effects of medication or undetected neurological conditions on perception or response processes (e.g., [19,20]). Our final sample, thus, consisted of 28 participants (24 females, *M* = 24.4 years old, age range:18–34). Participants had normal (or corrected-to-normal) sight and hearing and received course credit for their participation. The experiment was performed in accordance with the ethical standards laid down in the 2013 Declaration of Helsinki and according to the provisions of Greek law (4521/2018) given that the Ethics Committee at Panteion University of Social and Political Sciences came into force on 28 July 2021.

### Stimuli

The abstract sounds were identical to the ones used by Warren et al. [3]: a low tone (796 Hz), a high tone (1000 Hz), a hiss (2000 Hz broadband noise), and a buzz (40 Hz square wave). Each sound had a duration of 200 ms, with sampling rate 44.1 kHz and 32-bit coding. The environmental sounds utilized were selected after an online pilot study (12 participants were recruited, who did not participate in the main experiment). Participants were presented with 25 environmental sounds (retrieved from https://www.freesound.org and http://www.freesfx.co.uk) and they were asked to name all of them by providing one-word names. We selected the four with the highest rate of unanimity in terms of suggested names (see Table 1). All sounds were processed with the Audacity software [21].

**Table 1:** Environmental *sounds* with highest naming agreement (pilot study).

Sound	Most common name	Agreement rate (%)
Glass breaking	Break	58.33
Bird chirping	Whistle	75.00
Water drop	Drop	91.67
Dog barking	Bark	100.00

### Sound names

As previously described, participants reported the order of presented stimuli by successively clicking on screen buttons with the sounds’ names. For each participant, an online questionnaire was administered, one day before their visit at the laboratory, requiring them to provide one-word names for each sound. These idiosyncratic names were used for each participant and appeared as labels on the screen buttons during the ordering tasks. Analysis of participants’ responses showed that environmental sounds had greater agreement rate on selected names and fewer alternative names compared to abstract sounds (see Table 2), suggesting that the environmental sounds used in our study were indeed more nameable compared to the abstract sounds.

**Table 2:** Most common names given during the main experiment.

Sound	Most common name	Agreement rate	Sum of alternatives
*Environmental sounds*			
Dog barking	Dog	50%	5
Bird chirping	Whistle	35.70%	10
Water drop	Drop	53.50%	10
Glass breaking	Glass	57.10%	7
*Abstract sounds*			
Buzz	Bass	10.80%	21
High tone	Machine/Button/Beep	10.80%	19
Hiss	Interference	32.10%	13
Low tone	Phone	25%	20

### Experimental procedure

Participants sat in front of a desktop in a quiet room, safe from distractions. The experimenter entered the room only before each task, to provide instructions. At the end of each task, participants informed the experimenter and were asked if they needed a break. Before administering the first experimental task, the experimenter played the sounds presented at the questionnaire and reminded the participants of the names given by them for each sound. Participants took part in four tasks administered in succession: two enumeration tasks and two ordering tasks (Note: Task order was fixed for all participants. The ordering tasks always followed the enumeration tasks, so that participants would not guess the number of sounds based on the ordering tasks. The abstract sound condition always preceded the environmental sound condition to avoid fatigue as they were considered more difficult to complete.). In each enumeration task, all possible sequences of the four abstract (Task 1) and the four environmental (Task 2) sounds were presented in random order. At the beginning of each task, instructions were given, and four practice trials were administered. In each trial, a fixation cross appeared for 500 ms, the sequence was then presented with no interstimulus interval between the sounds (lasting in total 800 ms), and afterwards the participant was required to provide an answer to the question “How many sounds did you hear?” by pressing a numerical key on the keyboard (0–9), with no time out. The four sounds of each task constituted 24 possible sequences, which were presented 4 times each, totaling 96 trials per task. Past research has shown that participants can discern sounds individually when presented in a sequence of fewer than four [3]. Considering this, along with the specificity of the phenomenon first reported in Warren et al. [3] regarding difficulties in accurately recalling the sequence of four sounds, we decided to consistently present four sounds in all trials. Sequences were presented in random order. There was an optional short break after every 24 trials.

Similarly, in the ordering tasks all possible sequences of the four abstract (Task 3) and the four environmental (Task 4) sounds were presented in random order. At the beginning of each task, instructions were given, and four practice trials were administered. In each trial, a fixation cross appeared for 500 ms and the four sounds were presented as described for the enumeration tasks. The participants were required to respond to the question “In which order did you hear the sounds?”. The idiosyncratic sound names were presented as buttons on the screen, in a horizontal arrangement and in random order between trials. Participants were asked to click on each button name in the order they believed to have heard the individual sounds. The response screen remained until four clicks were provided (irrespectively of which buttons were clicked). Again, there was no time out. The number of trials and the breaks were identical to those described for the enumeration tasks. All tasks were implemented in OpenSesame [22].

## Results

### Enumeration tasks

**Fig 1:**
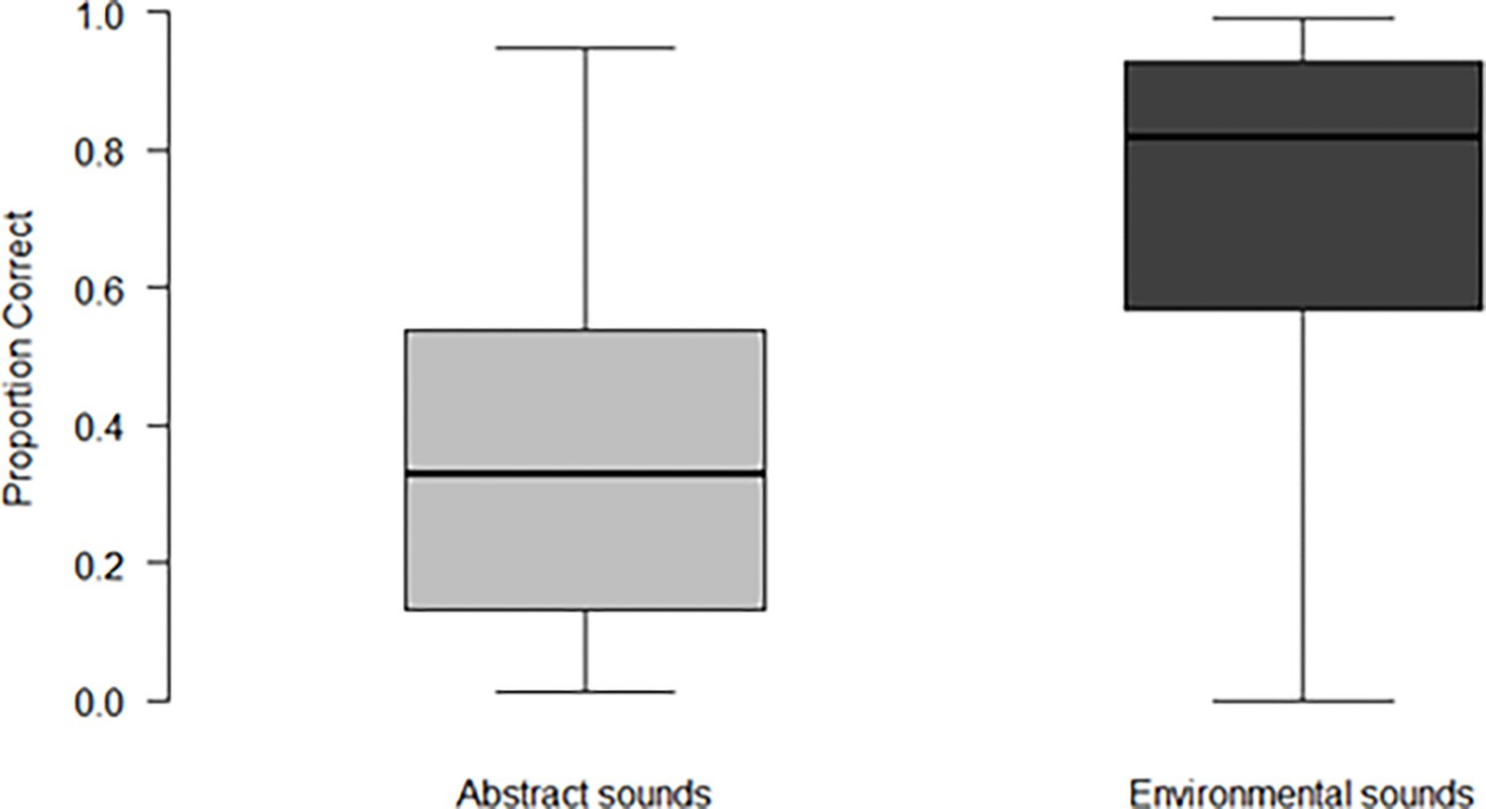
Enumeration task accuracy by sound category. *Note*. Boxes denote interquartile range; thick lines mark the median; error bars extend to the full range.

Participants’ accuracy in reporting the number of sounds presented in the abstract sounds task was 37.72% (*SD* = 27.8). Accuracy was higher in the environmental sounds task, reaching 67.97% (*SD* = 31.5; see Fig 1). Dependent samples t-test analysis showed that accuracy was different between the two tasks, *t*(27) = 4.74, *p* < .001, *d* = 0.1. We also examined if participants’ performance in each sound category was at chance performance (defined as 1/9, given that there were nine possible response keys). Participants’ accuracy both for the abstract and the environmental sounds was greater than chance, as revealed by one-tailed t-tests (abstract sounds: *t*(27) = 5.07, *p* < .001; environmental sounds: *t*(27) = 9.54, p < .001).

We used a one-way analysis of variance (ANOVA) for data collected from each task to investigate whether specific sequences of sounds (24 distinct levels) had any effect on accuracy [5]. Τhere was no effect of the specific sequence of sounds neither for the abstract task [*F*(11, 296.99) = 1.69, *p* = .07], nor for the environmental task [*F*(9.69, 261.72) = 1.31, *p* = .22.

#### Supplementary analyses of enumeration tasks

**Fig 2:**
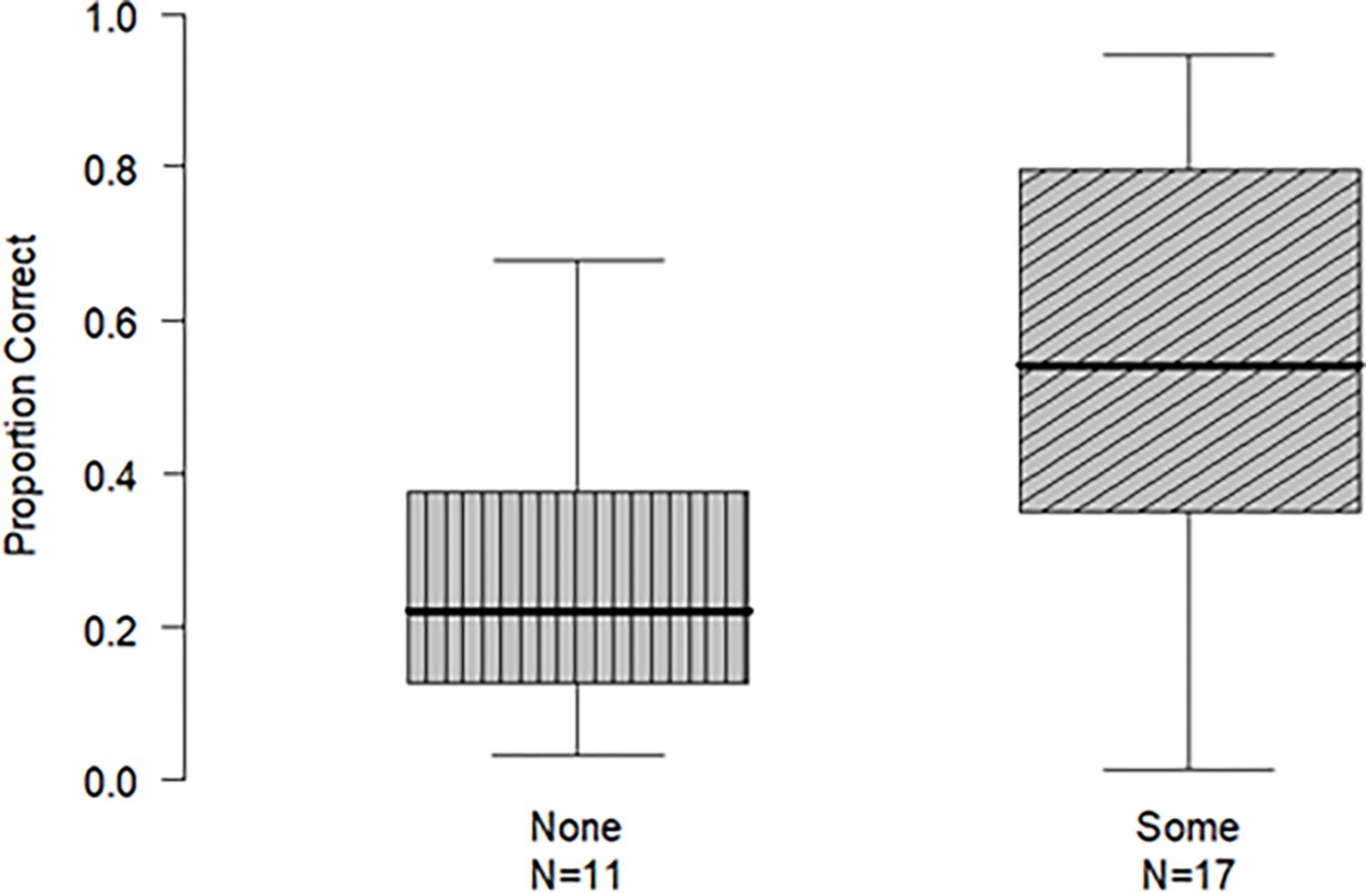
Accuracy of participants with and without music experience in the abstract enumeration task. *Note*. N denotes number of participants; boxes denote interquartile range; thick lines mark the median; error bars extend to the full range.

*Effects of musical training*. Graphical inspection of the data suggested that participants with musical training (with some experience but not over our exclusion criterion, N = 11) were more accurate compared to participants with no musical training (N = 17; see Fig 2). We, therefore, conducted a post-hoc analysis on accuracy for each counting condition, examining the influence of musical training, a factor with two levels: “some” vs. “none.” For the abstract sounds, participants with some musical training were more accurate (*M* = 55%, *SD* = 32) compared to participants with no training (*Μ* = 27%, *SD* = 18), as revealed by an independent samples t-test [*t*(14.226) = 2.61, *p* = .02]. For the environmental sounds, participants with some musical training had similar accuracy (*M* = 76%, *SD* = 28) with the group with no musical experience (*M* = 63%, *SD* = 33), as revealed by an independent samples t-test [*t*(26) = 1.10, *p* = 0.28].

*Absolute Error Analysis*. We also considered an alternative accuracy measure, namely the absolute error (henceforth AE) between participants’ responses and the correct response, since this measure might provide a more informative measure of participants’ accuracy, compared to the dichotomous correct/wrong typical accuracy score. Participants’ AE average score in the enumeration abstract task was 0.73 (*SD* = 0.39) whereas for the environmental sounds was 0.33 (*SD* = 0.34). A dependent samples t-test showed that this difference was significant [*t*(27) = 4.74, *p* < .001, *d* = 1.08].

We repeated the analysis of specific sound sequence (a within-subjects factor with 24 levels) on participants’ AE for both sound categories. For the abstract sounds, there was an effect of specific sound sequence on AE [*F*(11.12, 300.12) = 2.67, *p* = .003, *η*^*2*^ = 0.09]. Pairwise comparisons (Bonferroni corrected) revealed that participants’ AE differed between the sequences presented in Table 3. These differences failed to provide a clear pattern. For the environmental sounds, there was no effect of specific sound sequence on AE [*F*(9.05, 244,32) = 1.68, *p* = .094, *η*^*2*^ = 0.06].

**Table 3:** Significant differences of absolute error between sequence types in the abstract counting task.

Α sequence		Β sequence	Α-Β
Buzz-Hiss–High-Low	<	Hiss-Buzz-Low-High	= -0.26, *p* = .015
Buzz-High-Hiss-Low	<	Hiss-Buzz-High-Low	= -0.25, *p* = .048
Buzz-High-Hiss-Low	<	Hiss-Buzz-Low-High	= -0.28, *p* = .027
Hiss-Buzz-High-Low	>	Low-Hiss-Buzz-High	= 0.21, *p* = .012
High-Low-Hiss-Buzz	<	Hiss-Buzz-Low-High	= -0.31, *p* = .029

With respect to the effect of musical training, for the abstract sounds, participants with some musical training had an average AE of 47% (*SD* = 34) and participants with no musical training averaged 90% (*SD* = 34). This difference was significant [*t*(26) = 3.36, *p* = .003, *d* = 1.30]. For the environmental sounds, participants with some musical training averaged 24% (*SD* = 28) and participants with no musical training averaged 39% (*SD* = 37). This difference was not significant [t(26) = 1.18, *p* = .258, d = 0.47].

### Ordering tasks

**Fig 3:**
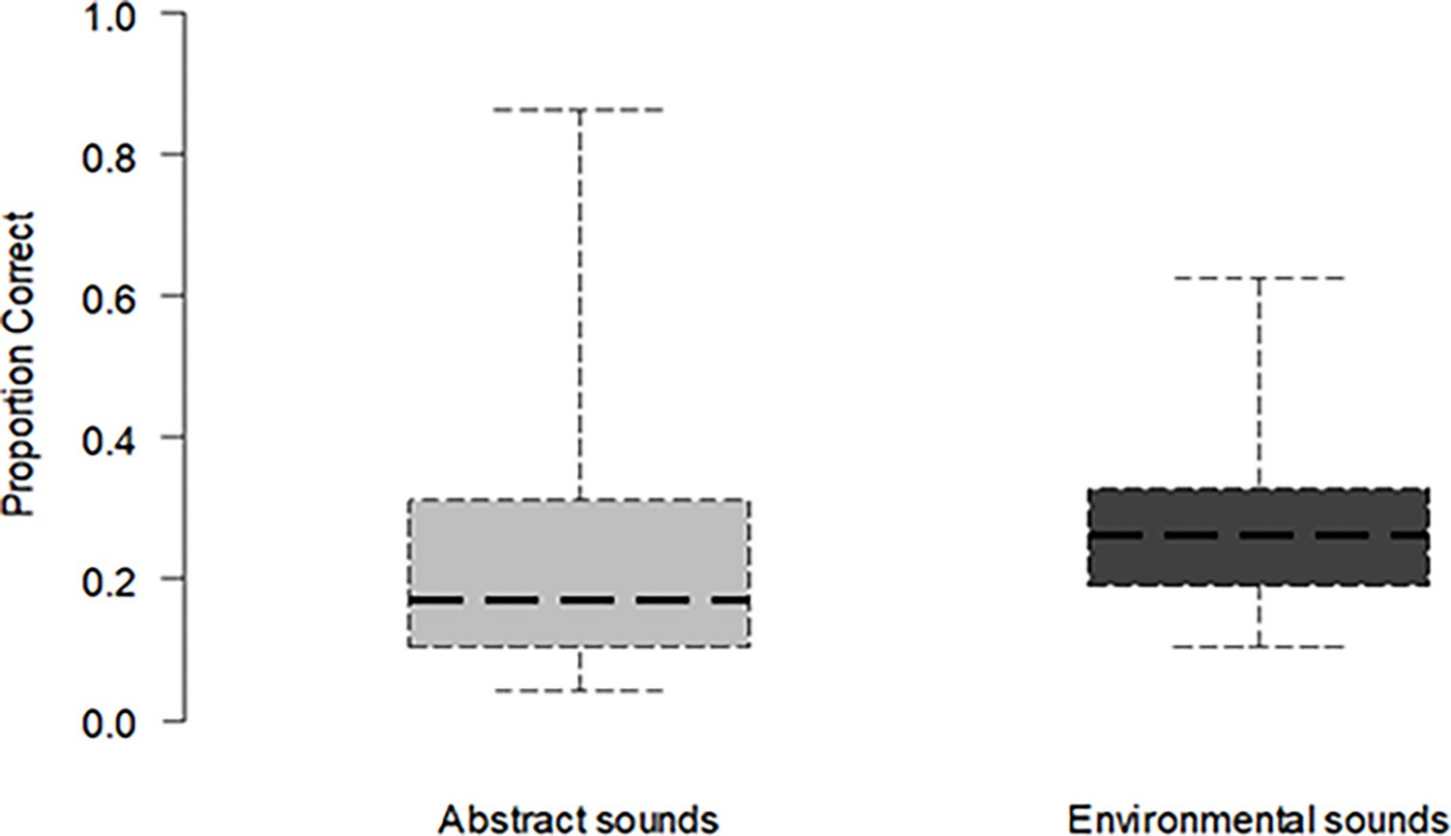
Ordering task accuracy by sound category. *Note*. Βoxes denote interquartile range; thick lines mark the median; error bars extend to the full range.

Participants’ accuracy in the ordering tasks was 23.4% (*SD* = 19.2) for the abstract sounds and 26.9% (*SD* = 10.6) for the environmental sounds (see Fig 3). This difference was not significant [*t*(27) = 1.54, *p* = .13, *d* = -0.23]. We also examined if participants’ accuracy was at chance levels (defined as 1/24, given that there are 24 permutations of the four buttons). Participants were found to exceed chance performance, both for the abstract and the environmental sounds, as revealed by one-tailed t-tests (abstract sounds: *t*(27) = 5.29, *p* < .001; environmental sounds: *t*(27) = 11.32, *p*< .001).

Similar to the enumeration tasks, we investigated the effect of specific sound sequences on order reporting accuracy. All post-hoc comparisons were Bonferroni corrected. For the abstract condition, a one-way ANOVA showed a main effect of sequence (with 24 distinct levels) on accuracy [*F*(23, 621) = 3.02, *p* < .001, η^2^*p* = 0.10]. Post-hoc multiple comparisons showed no significant differences of specific sequence types, suggesting that accuracy varied between all sequence types, but no specific difference emerged. For the environmental condition, there was also a main effect of sequence type (24 levels) on accuracy [*F*(23, 621) = 4.27, *p* < .001, η^2^*p* = 0.13] and post-hoc multiple comparisons showed seven significant differences (see Table 4). However, no clear pattern emerged, preventing us from providing a meaningful explanation for this result.

**Table 4:** Significant differences between sequence types in the environmental ordering condition.

Α sequence		Β sequence	Α-Β
Drop–Dog–Glass–Bird	<	Bird–Glass–Dog–Drop	= −.29, *p* < .001
Drop–Dog–Glass–Bird	<	Bird–Drop–Glass–Dog	= −.30, *p* = .011
Drop–Dog–Glass–Bird	<	Glass–Drop–Bird–Dog	= −.35, *p* = .001
Drop–Dog–Bird–Glass	<	Bird–Glass–Dog–Drop	= −.24, *p* = .016
Drop–Dog–Bird–Glass	<	Glass–Drop–Bird–Dog	= −.31, *p* = .008
Drop–Bird–Dog–Glass	<	Glass–Drop–Bird–Dog	= −32, *p =* .*006*
Bird–Dog–Drop–Glass	<	Glass–Drop–Bird–Dog	= −.29, *p* = .020

Moreover, we investigated whether the specific position that the sounds held in each sequence had any effect on the accuracy with which each sound was reported [5]. We, therefore, extracted reporting accuracy for each sound in a sequence and conducted a post-hoc one-way ANOVA of specific position (a categorical factor with four levels: 1^st^, 2^nd^, 3^rd^, and 4^th^ position) on accuracy. Again, all post-hoc comparisons were Bonferroni corrected. Results revealed a main effect of sound position, both for the abstract task [*F*(2.29, 64.54) = 59.14, *p* < .001, η^2^_*p*_ = 0.69], as well as for the environmental task [*F*(1.93, 52.35) = 181.73, *p* < .001, η^2^_*p*_ = 0.87]. Post hoc comparisons showed that for both tasks, the 1^st^ and 4^th^ sound was reported more accurately compared to the 2^nd^ and 3^rd^ sound. Similarly, accuracy for the 1^st^ sound was higher compared to the 4^th^, for both tasks.

**Fig 4:**
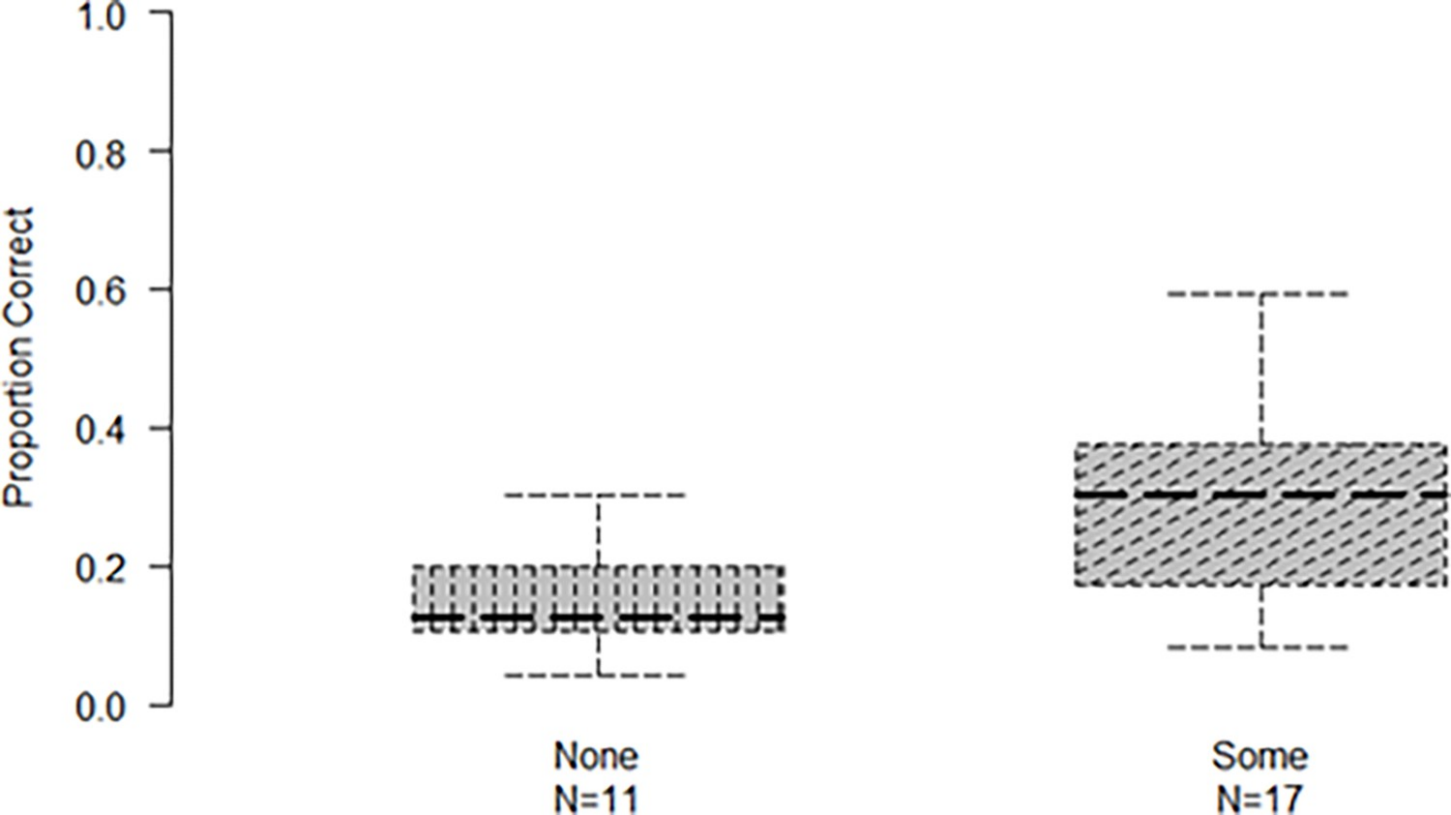
Accuracy of sound position by sound category in the ordering tasks. *Note*. Boxes denote interquartile range; thick lines mark the median; error bars extend to the full range.

The effect of increased accuracy for the first and last sound in the sequence did not seem to be the same across the two sound types (see Fig 4). To investigate this, we conducted a post-hoc two-way ANOVA with the within-subjects factors of specific sound position (1^st^, 2^nd^, 3^rd^, and 4^th^) and task (abstract vs. environmental) on accuracy for each sound. There was a main effect of task [*F*(1,27) = 13.51, *p* = .001, η^2^_*p*_ = 0.33] and a main effect of sound position [*F*(2.1, 56.74) = 174.24, *p* < .001, η^2^_*p*_ = 0.86]. Importantly, there was an interaction of position that each sound held with task [*F*(2.15, 57.93) = 30.46, *p* < .001, η^2^_*p*_ = 0.53]. The interaction between position and task might be attributed to the fact that in the environmental task the 2^nd^ sound was reported more accurately compared to the 3^rd^ (difference in accuracy: *M* = 4.4%, *SD* = 6.1, *p* = .005), something that was not observed in the abstract task (difference in accuracy: *M* = 2%, *SD* = 6.7 *p* = .78; see Fig 4). To further investigate whether the position by task interaction could also be due to the differences in accuracy between successive sounds for the two sound categories, we also conducted multiple comparisons of successive differences. Results showed that the *difference* in accuracy between the 1^st^ and 2^nd^ sound was greater for the environmental sounds as compared to the abstract sounds [*t*(81) = 7.55, *p* < .001]. Additional multiple comparisons of accuracy between abstract versus environmental sounds per sound position (1^st^, 2^nd^, 3^rd^, and 4^th^) revealed a significant difference: Participants were more accurate in reporting the first environmental sound (*M* = 80.32%, *SD* = 12.39) compared to the first abstract sound (*M* = 60.9%, *SD* = 19.47) [*t*(27) = −6.42, *p* < .001]. For the second, third, and fourth sound position there was no difference between sound category (all *p*s>.05).

### Supplementary analysis of ordering tasks

**Fig 5:**
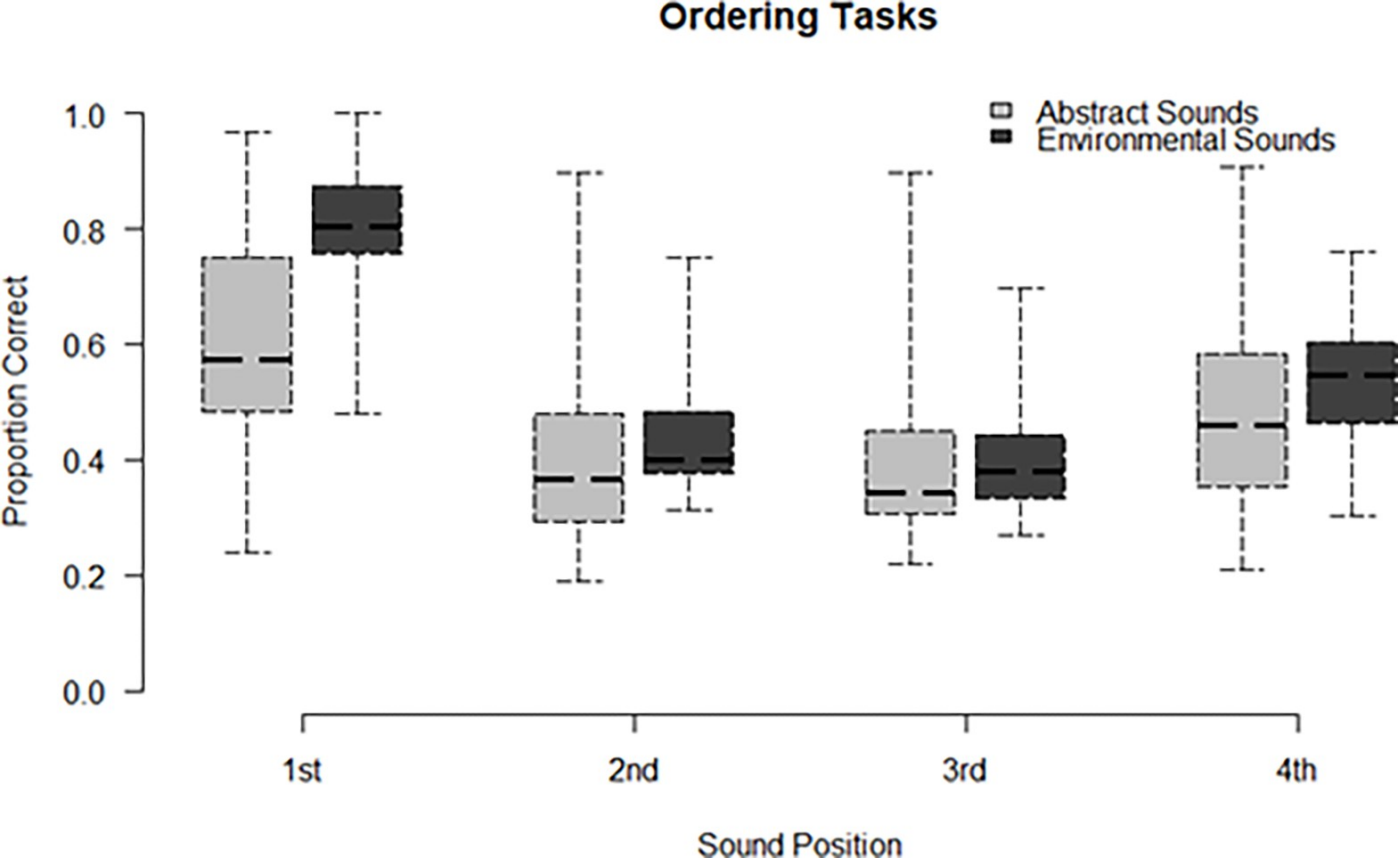
Accuracy of participants with and without music experience in the abstract ordering task. *Note*. N denotes number of participants; Boxes denote interquartile range; thick lines mark the median; error bars extend to the full range.

*Effects of Musical Training*. Again, we contrasted accuracy in reporting the order of sounds of participants with some musical training and those with no musical training. For the abstract sounds, participants with some musical training exhibited higher accuracy (*M* = 32%, *SD* = 23) as compared to participants with no training (*M* = 17.5%, *SD* = 23.3) [*t*(26) = 2.11, *p* = .044, *d* = 0.77] (see Fig 5). For the environmental sounds, accuracy of participants with some musical training (*M* = 28%, *SD* = 13) did not differ from the accuracy of those with no musical training (*M* = 25%, *SD* = 8) [*t*(26) = 0.59, *p* = .55].

## Discussion

In the present study, we re-examined the assumption that nameability affects perception of temporal order of brief auditory stimuli [11,13], by contrasting participants’ accuracy in order-reporting tasks between two sound categories: easily named stimuli (environmental sounds) and hard-to-name stimuli (abstract sounds). We also administered enumeration tasks to investigate whether participants perceived and identified the stimuli presented. Environmental sounds were found to be enumerated more accurately compared to abstract sounds. Our results showed that participants exhibited equally low accuracy for both environmental and abstract sounds, suggesting no effect of stimulus nameability on overall order perception. Nameability of the stimuli, however, influenced both the enumeration and ordering tasks. The effect was clear in the enumeration tasks, where accuracy differed significantly between the two conditions, but in the ordering tasks the effect of nameability was evident only in the per-position accuracy between abstract and environmental sounds. The effect of nameability, however, was not strong enough to amplify overall order perception, contrary to the prediction that it would enhance it [13], and contrary to the findings initially reported by Warren et al. [3], where nameability seems to be a factor strongly associated with order perception of consecutive sounds (each lasting 200 ms).

In the enumeration tasks, our results suggested an effect of nameability. If we assume that the enumeration tasks we utilized recruit memory processes, the difference in counting accuracy between environmental and abstract sounds corroborates observations by Bartlett [23] and Bower and Holyoak [24], who posited that verbalization of stimuli affects free recall and recognition performance. Similarly, Warren [11] suggested that counting stimuli depends on attaching verbal labels to them upon presentation. Further, Edworthy and Hards [25] suggested that retention of a sound name depends on the ease with which the sound can generate multiple cues. Specifically, when participants generate their own labels for a stimulus, they do so in a way that it is diversly encoded (i.e., they will use verbal labels that lead to representative images). Most often, the labels will be the object or event generating the sound, which is more plausible for natural stimuli. These claims further support the notion that naming fluency facilitated free recall and counting of the environmental sounds in our study. Regardless, though, of the difference in accuracy between sound categories, it is important to note that accuracy was low for the counting tasks of both sound categories (abstract sounds: *M* = 37.72%, *SD* = 27.8; environmental sounds: *M* = 67.97%, *SD* = 31.5). This result suggests that participants did not always perceive and individuate the stimuli presented. Interestingly, this difficulty emerged even though participants were presented with the sounds and names they had provided right before executing the tasks. Nevertheless, results remained above chance level and, hence, interpretable.

In the ordering tasks, there was an effect of nameability only when comparing per-position accuracy. Specifically, in both abstract and environmental sequences, the first and last sounds of a sequence were reported more accurately than the middle sounds. However, analysis also showed an interaction of sound position with sound category. This interaction is attributed to the finding that between the two sound categories (i.e., abstract, environmental), accuracy fluctuated between positions in different ways: the difference of accuracy between the first and second sound was greater for the environmental sounds as compared to the abstract sounds. In other words, our results suggest that the first in a stream of sounds is indeed better reported than the middle sounds and that this enhanced performance is further affected by stimulus nameability. In sum, there seems to be a qualitative difference in the processing of environmental versus abstract sounds. We suggest that further research is required to replicate these results and examine possible factors driving this qualitative difference, given that the distinction of sounds based on their nameability has been used in literature ([3]; see also [26,27] for similar manipulations of nameability of stimuli in the visual modality), without accounting for other possible differentiation factors, such as the amount of information in each category.

The low accuracy in the ordering tasks of the environmental sounds seems to contrast the results of Warren et al. [3]. This discrepancy might be attributed to differences in methodology, as well as in material selection. The methodological differences compared to Warren et al. are that, firstly, we implemented a computerized collection of answers. Secondly, participants provided their own names for the sounds, and thirdly, in the current study, each sequence was presented once, and each participant took part in multiple trials. All these methodological differences could account for the non-replication of results. We also consider the issue of material selection as of crucial importance for the specific study. Warren et al. contrasted order-reporting accuracy between abstract sounds and verbal stimuli (i.e., spoken digits: “one”, “three”, “eight”, “two”), even though perception of verbal stimuli could be mediated by other, language-related processes. We, on the other hand, contrasted order-reporting accuracy between abstract sounds and environmental, non-verbal sounds. It could be the case that greater familiarity with the spoken digits used in Warren et al. [3] is the primary source of this difference. Warren and Ackroff [12] discussed the possibility that for stimulus durations up to about 200 ms, perception of order can be achieved by HPR, whereby a sequence of sounds is first perceived as a whole, based on changes in quality and pitch. Subsequently, they argued, this sequence gets broken down to its comprising parts. Importantly, they argue that this compartmentalization can only be successful if participants are familiar enough with the individual stimuli of a sequence, so as to infer the order of presentation to produce the total sequence. Given that our environmental sounds were selected in a pilot study on the basis of name agreement (which was further supported by the names participants provided in an online questionnaire), one might argue that the pattern of the present results is not consistent with HPR processes and cannot support the use of HPR processes for non-related sounds (i.e., sounds that constitute neither music nor speech).

Warren and Ackroff [12] also suggested an alternative mechanism of order perception through naming, Direct ICO. Since no difference in ordering accuracy of four sounds emerged from our manipulation and given the fact that participants’ labels for the environmental sounds were more unanimous compared to those for the abstract sounds, this mechanism, arguably, also failed to mediate order perception in the specific paradigm. It could be argued that Direct ICO can only be used for stimuli of greater durations than the duration of the stimuli presented in this study. As neither HPR nor Direct ICO seem to account for the perception of the stimuli used in the present study, we could hypothesize that order perception at this presentation rate might be more accurate for more familiar stimuli such as the spoken digits used by Warren et al. [3], where the stimulus coincides with its name. It could be the case that non-linguistic sounds at this presentation rate exceed our processing capacity, implying a limitation in memory processes, or that processing at this presentation rate is facilitated by language. Verbal stimuli could help expand order processing ability to higher presentation rates. For example, people with Specific Language Impairment (SLI) have accompanying deficits in order perception of non-verbal stimuli, needing lower presentation rates to perform as accurately as healthy individuals [28]. Theories on the origins of SLI pose a fundamental non-verbal deficit of auditory perception, which results to language impairment [29]. It seems that advanced auditory perception is a prerequisite for language acquisition, while language can then be used as a means for further supporting auditory perception. This idea of a basic-level interaction of language with other cognitive processes lies in the core of the Whorfian questions (e.g., [30,31]. We, thus, argue that our investigation may be considered important both for practical and theoretical reasons. That is, both for research on language deficits and our understanding of the relation between language and purportedly non-linguistic perceptual processes.

Our finding that participants were more accurate in reporting the order of the first and last sound in a sequence as compared to the middle two sounds was first reported by Warren [6] and is also in alignment with the work of Chun [8]. He showed that in a series of rapid serial visual presentation tasks, after locating a target stimulus, subsequent targets that appeared within the next 500–600 ms, following distractors of 100 ms, were located with significantly lower accuracy. Similarly, Vatakis et al. [32] reported that in successive presentations of asynchronous audiovisual targets, participants were better at perceiving the order of succession when stimuli were placed at the beginning or the end of the stream rather than the middle of the sequence. Thus, it is reasonable to assume that this middle stream accuracy “deficit” might be due to a limitation of short-term memory capacity in ordering tasks. However, our results also showed low accuracy at the counting tasks, as well. Even though there was an effect of sound category, accuracy was not very high, with mean counting accuracy reaching 68% for the environmental sounds. Thus, it seems that our participants did not always perceive all the sounds presented at each trial. Our results, therefore, may suggest that when we have stimuli presented at these rates there is some limitation in the mere perception of the number of stimuli presented, let alone in reporting their succession (nevertheless, performance in the ordering tasks was above chance level). This contradicts previous findings such as those of Song and Luo [33], who propose that memory for sounds is organized at temporal chunks of 200 ms. Also, we cannot be certain whether better identification of the first stimulus leads to worse identification of the following stimuli—an “attentional blink phenomenon” [8]—or whether these two are independent.

It is also quite interesting that our explorative post-hoc analysis showed that participants with some experience of musical training exhibited higher accuracy for the abstract sounds for both enumeration and ordering tasks, whereas the two subgroups (with or without musical experience) did not differ when it came to environmental sounds. As mentioned in the Methods section, we included in our sample participants that had less than 10 years of musical training, and who did not in the time of the experiment practice music. However, we discarded data from participants that had more than 10 years of experience and were currently practicing music. This decision was based on the heterogeneity and diverse criteria of the definitions of ‘expert musicians’, ‘amateurs’, and ‘non musicians’ in existing literature [34]. For instance, some studies have taken into account hours of daily training, age of training onset, and total years of practice and do not necessarily control for the complete absence of musical experience in the control groups (e.g., [35,36]), Other studies have defined “musicians” as people with more than 10 years of training (e.g., [37]), or less than 10 years but have also used the criterion of continuous daily practice [38,39]. We, therefore, did not have strong reasons to a-priori consider any length of musical experience as a confounding factor. Nevertheless, we kept the threshold of 10 years, since those participants were trained on a professional level (i.e., had music diplomas) or were still practicing music.

Warren and Obusek [5] chose these specific sounds (the high and low tone) to investigate whether the melodic combination of the two tones would help participants to perceive the order of the stimuli presented. However, it is possible that in our study participants had difficulty in recognizing which name (given by them) matched to which tone. Indeed, many participants mentioned a difficulty in recognizing the tone matching to each name. Furthermore, during the debriefing, some participants mentioned that they misinterpreted the two tones by unifying them as one. Nevertheless, our analysis did not show decreased performance in sequences where the two tones were in succession. However, we found that participants with music experience had significantly better performance in the abstract condition. Those might have had higher accuracy due to increased familiarity with artificial sounds or because they have been trained in recognizing similar sounds (notes) of different frequency. Rammsayer and Altenmüller [40] argue that people with professional music training (minimum 14 years) have more accurate temporal perception than non-musicians in tasks that rely on short-term memory. Our results suggest that temporal short-term memory enhancement might be true for participants that are not professional musicians with some musical experience. Although this pattern of results was not predicted, we argue that it speaks of the importance of the nature of the sounds, and the familiarity participants may have depending on their training. The factor of musical training, we argue, should be a controlled or manipulated factor in subsequent studies of serial order perception when examining auditory processes. We also suggest, that to better understand order perception processes, future studies should not include stimuli that are easy to confuse either because of their similarity or their melodic properties.

Taken together, our results indicate that with stimuli lasting 200 ms, participants failed both in reporting the order of sounds presented in succession, and, importantly, also failed to perceive the number of sounds presented. Even though there was an effect of sound category in the enumeration tasks, participants did not seem to correctly perceive the number of sounds presented at each trial. This finding could have implications for studies using rapid presentation of stimuli. We should highlight the fact that order perception of environmental stimuli seems to be accomplished through qualitatively different processes compared to the ones mediating order perception of abstract stimuli. Also, research is needed to conclude whether higher accuracy for initial and/or final stimuli in a stream leads to lower accuracy in midstream stimuli or whether those two possibilities are independent. Moreover, our results suggest that musical training seems to affect counting and order perception accuracy of abstract stimuli. We also discuss the role of previously used tones in this area of research and suggest that tones forming melodic sequences should be avoided in future experiments.
